# Supplementary material for: HDAC6 and ERK/ADAM17 Regulate VEGF-Induced NOTCH Signaling in Lung Endothelial Cells
Source: Cells. 2023 Sep 8;12(18):2231. doi: 10.3390/cells12182231 (PMC10526732; doi:10.3390/cells12182231)
Supplement: Supplementary file 1 [file cells-12-02231-s001.zip › cells-2533020-supplementary.pdf]

Supplementary Figure S1

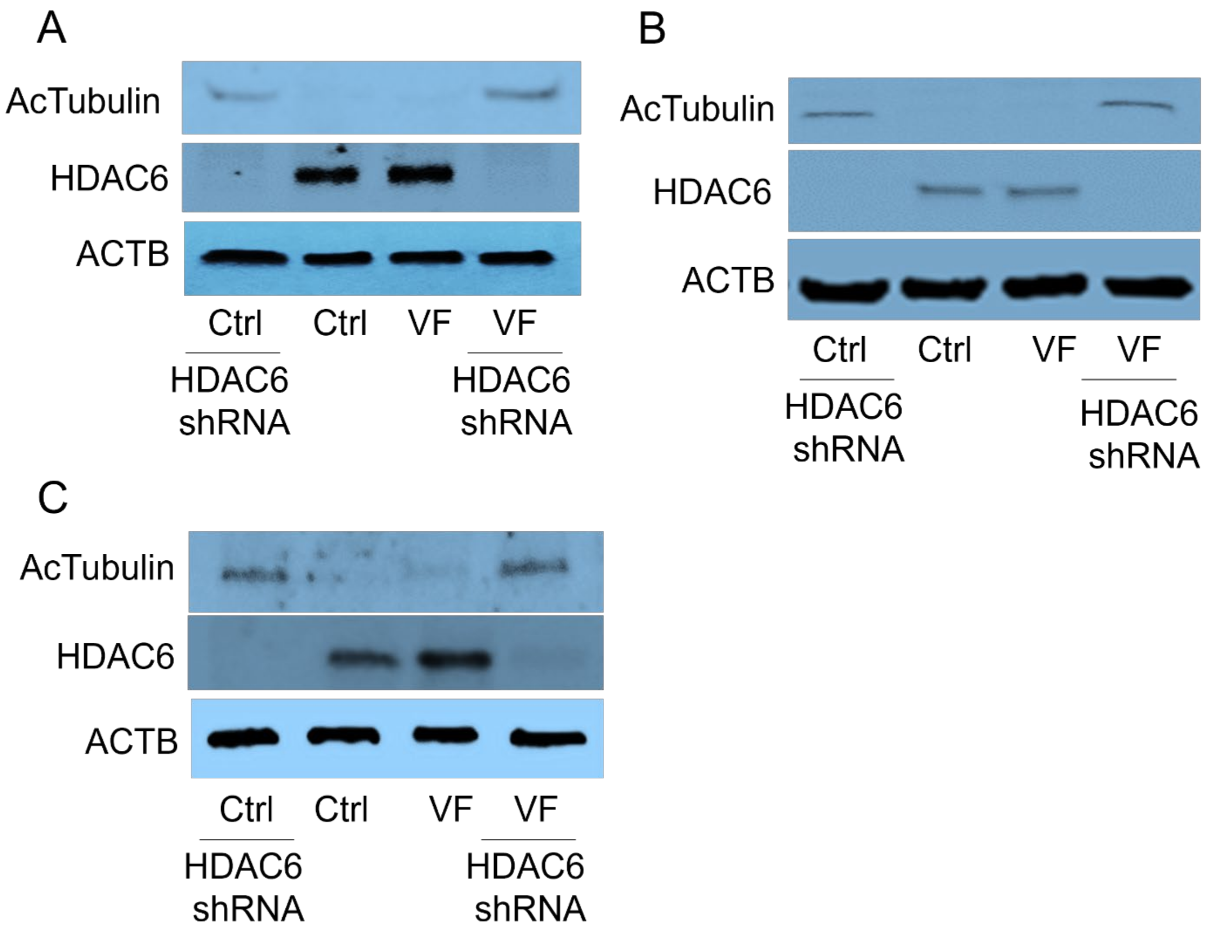

Supplementary Figure S1. HPMEC-Im transduced with lentivirus containing sc shRNA or HDAC6 shRNA were treated with 50 ng/ml VEGF for 45 minutes. Cell lysates were partitioned for western blot analysis to determine efficiency of HDAC6 silencing (shown here) and corresponding co-immunoprecipitation studies shown in Figure 4A **(A)**, Figure 4C **(B)**, and Figure 4E **(C)**.
